# Improved Microbial Safety of Direct Ozone-Depurated Shellstock Eastern Oysters (*Crassostrea virginica*) by Superchilled Storage

**DOI:** 10.3389/fmicb.2018.02802

**Published:** 2018-11-22

**Authors:** Karla López Hernández, Violeta Pardío Sedas, Sóstenes Rodríguez Dehaibes, Víctor Suárez Valencia, Isaura Rivas Mozo, David Martínez Herrera, Argel Flores Primo, Roxana Uscanga Serrano

**Affiliations:** ^1^Doctorate Program in Agricultural Sciences, Facultad de Medicina Veterinaria y Zootecnia, Universidad Veracruzana, Veracruz, Mexico; ^2^Facultad de Medicina, Universidad Autónoma de Coahuila, Saltillo Coahuila, Mexico

**Keywords:** American oyster, direct ozone depuration, superchilled storage, *Vibrio cholerae*, *Salmonella* spp., *Escherichia coli*, fecal coliforms

## Abstract

The effect of superchilled storage at -1°C on the microbial safety of oyster depurated with 0.2, 0.4, and 0.6 mg/L ozone was studied for 14 days. Fecal coliforms (4,100–16,000 MPN/100 g), *Escherichia coli* (1,500–3,650 MPN/100 g), *Vibrio cholerae* non-O1/non-O139 (13.0–102.0 MPN/g), and *Salmonella* spp. (2.270–3.035 × 10^3^ CFU/g) were initially present in raw oysters. After 6 h depuration, fecal coliform counts decreased (*P* < 0.05) to 300, 20 and 20 MPN/100 g for 0.2, 0.4, and 0.6 mg/L treatments, while a 0.3 log decrease in control oysters was observed. Initial *E. coli* counts decreased (*P* < 0.05) in oysters to 50, 20, and 20 MPN/100 g for 0.2, 0.4, and 0.6 mg/L treatments, respectively. A 1 log reduction in *V. cholerae* non-O1/non-139 levels were observed in 0.4 and 0.6 mg/L-treatments after 2 and 4 h depuration. *Salmonella* spp. was not detected in oyster samples after 6 h depuration in 0.4 and 0.6 mg/L-ozone treatments. Considering the bacterial loads after depuration, at the end of superchilled storage the 0.4 mg/L-ozonated oysters attained lower (*P* < 0.05) fecal coliform levels (280 MPN/100 g) and *E. coli* counts in 0.4 and 0.6 mg/L-ozonated oysters (20 and 95 MPN/100 g, respectively). A 2-log decrease in *V. cholerae* non-O1/non-O139 levels on day 5 in 0.4 and 0.6 mg/L-ozonated oysters (< 0.3 MPN/g) was attained. *V. cholerae* non-O1/non-O139 counts in control oysters decreased 1 log on day 9 of superchilled storage. *Salmonella* spp. was not detected in ozonated and superchilled stored oysters. Levels of fecal coliforms, *E. coli, Salmonella* spp., and *V. cholerae* non-O1/non-O139 in non-ozone depurated oyster samples were higher than in control, 0.4 and 0.6 mg/L ozonated oyster samples during superchilled storage. The cumulative mortality rates after 14 days of storage for superchilled oysters (22.2%) was higher (*P* < 0.05) than 0.6 mg/L O_3_ (7.2%) and 0.4 mg/L O_3_ (5.8%) treatments, and control oysters (5.6%). pH values in control oysters decreased significantly (*P* < 0.05) throughout the storage period but not in oysters of both ozone treatments, indicating no detrimental effects on oyster survival. The results of this study suggest that superchilled storage enables ozonated shellstock oysters (0.4 mg/L-6 h) stored for 9 days to be safe human consumption.

## Introduction

One of the main concerns of the seafood industry is the health risk associated with traditional consumption of oysters, as most bivalves are eaten raw or minimally cooked, and whole, including the viscera. Besides being highly perishable, oysters can pose hazards to public health because their filter feeding behavior leads to the accumulation of pathogens naturally present in growing areas contaminated with polluted water, making the bivalves a high-risk food group (Pardío-Sedas, 2015). To improve their microbiological safety, raw mollusks are often depurated by placing them in controlled, sanitized seawater allowing them to purge contaminants from their tissues by means of their natural pumping activity. The effectiveness of the depuration process depends on the diversity and physiology of the particular mollusk species as well as on water and system characteristics (Schneider et al., 2009). Depuration systems consist of flow-through, or closed, recirculation with chemically (chlorine and/or ozone) or physically (UV irradiation) disinfected water to eliminate bacteria and other pathogens and spoilage microorganisms. The advantage of closed systems is that they use considerably less water than open systems.

Ozone has been given GRAS (Generally Recognized as Safe) approval by the USDA and the FDA for direct contact with food products (United States Food and Drug Administration [USFDA], 2016). Ozone acts on bacteria by oxidation of membrane glycoproteins and/or glycolipids, inhibition of membrane-bound enzymes, and it may also damage DNA due to oxidation of double bonds by singlet oxygen (O’Donnell et al., 2012). Depuration with ozonated water is a postharvest process for shellfish moderately contaminated with fecal coliform bacteria so as to increase the availability and supply of safe, nutritious bivalves such as oysters. However, ozone-depuration efficiency of mollusks with high initial levels of *Escherichia coli* and *Vibrio* spp. could lead to insufficient reduction of pathogen loads. Since different microorganisms respond differently to the depuration process, it may fail to guarantee consumer safety (Croci et al., 2002; Food and Agriculture Organization [FAO], 2008). A growing body of research has identified direct application of ozone as a beneficial technology, defined as direct application of residual ozone and ozone-produced oxidants to farmed species of finfish and shellfish species inside the recirculating systems, and is thus distinct from conventional discrete ozone usage. This approach appears to be increasingly employed due to proven enhancement of hygiene, water quality and production, provided dosages are appropriate to maintain animal health and welfare. Appropriate ozone dosages varied from 200 mV up to 600 mV (Powell and Scolding, 2018). The chemistry of ozone in seawater is considerably different from that in brackish and freshwater. When seawater is ozonated, the ozone reacts with a variety of naturally occurring chemical species. The most important reaction is the oxidation of bromide ions (Br^-^), forming hypobromite ions (OBr^-^) (Oemcke and van Leeuwen, 1998). In seawater with a typical pH of 8, hypobromous acid (HOBr) will predominate and be the most important disinfectant with a half-life of hours to days depending on light conditions and water quality characteristics. Thus, continuous ozonation is beneficial because seawater quality for depuration remains relatively stable (Summerfelt et al., 2009). During depuration, specific water parameters such as pH, temperature and salinity, need to be applied for each species, as changes in these parameters may cause shellfish to reduce or stop activity, thus reducing the effectiveness of the depuration process (Lee et al., 2008). Even though ozone may become less soluble and less stable as temperature and pH increase during depuration, the resulting radical species contribute to its efficacy.

Although the depuration process appeared to be a promising postharvest treatment for minimizing risks of infections associated with raw oyster consumption, it would be necessary to use this in conjunction with other inactivation treatments to achieve better decontamination efficacy. Combined post-harvest techniques have been also studied, such as heat/cool pasteurization (Andrews et al., 2000), high hydrostatic pressure (Ahmadi et al., 2015), and rapid freezing with frozen storage (Liu et al., 2009). However, bivalves die during these processes. Ozone treatment has been proven effective for slowing down the reproduction of spoilage bacteria in mussels, especially when combined with refrigeration (Manousaridis et al., 2005). Nevertheless, efforts to minimize foodborne illnesses through proper refrigeration of postharvest oysters may be compromised as *Vibrio cholerae* strains exhibit significant differences in survival rates in artificially contaminated seafood at refrigeration or freezing temperatures (Johnston and Brown, 2002). Among the different refrigeration preservation techniques, superchilling technology is one of the most efficient and promising technologies for storing raw seafood. Superchilled food products are stored 1–2°C below the initial freezing point of the product, just below -1.0°C under closely controlled conditions to extend the seafood shelf-life at least 1.4∼4 times that of traditional chilling (Magnussen et al., 2008). In the range of superchilling temperature (0 to -4°C), microbial activity is inhibited, and most bacteria are unable to grow. Accordingly, reduction in microorganism growth rate is due to a synergistic effect of the reduction in water activity and temperature (Kaale et al., 2011). In order to prolong the shelf life of aquatic foods, the effect of superchilling in conjunction with other techniques has been studied, promising preservative potentials in reducing microbiological contents. Sequential application of certain set of preservative factors or hurdles has been shown to result in a higher level of inactivation than the sum of inactivation levels achieved when each preservative is applied separately. This enhanced inactivation is often referred to as a synergism. Synergism is beneficial since the disinfectant dose and the reaction time required for the same level of inactivation can be reduced. A synergistic effect could be achieved if the hurdles in a food hit different targets (e.g., cell membrane, DNA, enzyme systems, etc.) within the microbial cells and thus disturb the homeostasis of the microorganism present (Leistner, 2000). In this regard, refrigeration can be combined with the use of ozone, therefore harnessing the results by a synergic effect (Gonçalves, 2009).

A few studies have reported that refrigeration or superchilling combined with ozonized water have a remarkable effect in reducing the microbial levels in seafood. Manousaridis et al. (2005) reported a shelf-life of 11–12 days for shucked vacuum-packed and refrigerated mussels (4 ± 0.5°C) ozonated 90 min in an ozone-saturated aqueous solution (1 mg/L). Our previous assays carried out during the windy season indicated that direct ozone depuration (0.2–0.6 mg/L) significantly reduced low levels of *E. coli* and the isolation of *V. cholerae* in oysters during the superchilled storage period. Biochemical analysis at 0.4 mg O_3_/L depuration, indicated no significant (*P* > 0.05) changes in the LYS levels of oyster meat after 6 h depuration. However, panelists detected a significant (*P* < 0.05) decrease in firmness, but not in elasticity (Pardío et al., 2010). Likewise, TBA values of 0.4 mg O_3_/L depurated oyster samples (2.629 MA/kg) were slightly higher (*P* < 0.05) than control samples (2.244 MA/kg), but no unpleasant flavors were detected (Rivas Mozo, 2010). Rey et al. (2012) reported that storage of oyster (*Ostrea edulis*) in ozonated slurry ice (0.2 mg/L ozone) at 0°C ± 2°C provided better control of *Enterobacteriaceae* than non-ozonated flake ice. Jianbing et al. (2013) reported that superchilling (-1.2°C) combined with ozonized water (1.8 mg/L) decreased the aerobic plate count of pomfret fillets. Recently, Bono et al. (2017) found that superchilled storage at -1°C improved the antimicrobial activity of 0.3 mg/L-ozonized slurry-ice during European anchovy (*Engraulis encrasicolus*) and sardine (*Sardina pilchardus*) postharvest preservation. However, to our knowledge, no information is available regarding the effect of ozone depuration combined with superchilled storage on *E. coli, Salmonella* spp. and *V. cholerae* survival in bivalves.

In Mexico, oysters are harvested extensively within the oyster-producing areas found along the Mexican Gulf coast. In Mexico, the state of Veracruz is the primary oyster producer, harvesting 26,713 tons annually, which accounts for 43% of the national average annual production (61,996 t) (Comisión Nacional de Acuacultura y Pesca [CONAPESCA], 2017). The American oyster (*Crassostrea virginica*) is one of the most popular bivalve mollusks, widely consumed in large quantities. They are sold alive in whole shell, shucked in fresh form or packaged and refrigerated in polyethylene bags. Although previous studies have revealed a high prevalence of *E. coli, V. cholerae* O1/O139 and *V. cholerae* non-O1/non-O139 *chxA* in oysters (*C. virginica*) harvested from estuarine lagoons in Veracruz (Pardío et al., 2010; López et al., 2015), a relatively high proportion of Mexican oysters sold in restaurants and markets is not currently subjected to any post-harvest process. Moreover, along the traid chain these specimens are not maintained in refrigerated conditions and are thus a health hazard. According to the Mexican Norm NOM-242-SSA1-2009 (Secretaría de Salud [SSA], 2009), that provides guidelines for the sanitary control and commerce of shellfish in Mexico, shellstock oysters should be kept alive and adequately refrigerated to an internal body temperature of 7°C and stored 7 days at most to ensure safe consumption. Because of the importance of raw oysters in gastronomy and economics, the improvement of their microbial safety is of major interest. Given the limited data available regarding the microbiological safety of superchilled ozonated shellstock oysters, the aim of this study was to determine the antimicrobial efficacy of superchilled storage on *V. cholerae*, fecal coliforms, *E. coli* and *Salmonella* spp. loads in direct ozone-depurated shellstock American raw oysters and compared with superchilled alone, as a cost-effective postharvest process for effectively reducing contamination without impairment of oyster viability, while ensuring public health.

## Materials and Methods

### Oyster Collection and Handling

The Project of which this study was part, was evaluated and approved by the Mexican National Council of Science and Technology CONACYT. The protocol was in agreement with the Mexican Official Norm MNX-FF-001-SCFI-2009 for fishery products and shellstock oysters. A total of 900 naturally polluted medium legal-size (7–8 cm long) (Secretaría de Economía [SE], 2011) live American oysters (*C. virginica*) were harvested on the first day of each experiment by divers during the dry season on several production beds in the Mandinga Lagoon System (MLS), Veracruz, México, located in the central region of the state of Veracruz. The oyster samples were immediately transported to the laboratory in coolers at 4°C; dead animals were discarded, and the remaining oysters were scrubbed and rinsed under high pressure tap water to remove debris and fouling organisms before being transferred to the depuration system tank for acclimatization. Duplicate samples of 40 raw oysters each (oysters without ozone treatment) were analyzed to determine the initial loads of fecal coliforms, *E. coli, V. cholerae*, and *Salmonella* spp. within 2 h of collection.

### Oyster Depuration Trials

**FIGURE 1:**
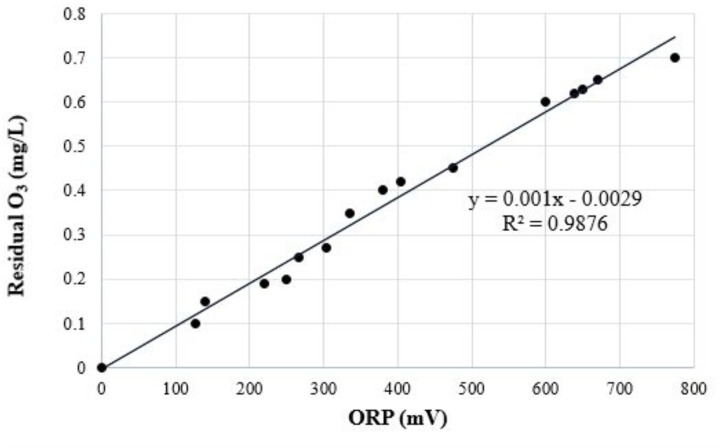
Plot of the mean dissolved ozone concentrations vs. mean oxidative reduction potential (ORP) measurements that were logged at the end of the ozone contact chamber. A linear regression shows the relationship between dissolved ozone and ORP.

Each depuration trial was carried out using one control tank filled with untreated artificial seawater (ASW) and two treatment tanks (units of replication) filled with ozonated ASW. ASW was ozonated in three sequential and independent trials: the first at 0.2, the second at 0.4, and the third at 0.6 mg/L. Ozone concentration was kept low in order to minimize bromate production. ASW was prepared by mixing a synthetic salt brand (Instant Ocean Salt - Aquarium systems, Inc., Mentor, OH, United States) with water purified by a reverse osmosis system, following the manufacturer’s instructions. Prior to each depuration trial, fecal coliforms and *E. coli* levels were monitored in duplicate ASW samples to establish a reliable starting point. To assist efficacy, cost and safety, ozonated ASW was produced by injecting ozone into a closed ASW chamber using a Venturi tube and then transferred directly into each treatment tank recirculated with a 1/5 HP pump (Boyu model FP-1100B, Guangdong Boyu Group CO., LTD., Chaozhou, China). The system adjusted the ozone output to match the ozone demand of the system. When the desired ozone dose was reached, the ozone generator was stopped to begin the process, and the pump continued cycling the ozonated ASW. Ozone was controlled and regulated automatically by a digital sensor oxidation reduction potential (ORP) controller (American Marine, Inc., Ridgefield, CT, United States) (Buchan et al., 2005), which was located at the end of the O_3_ contact chamber. ORP probe calibration required probe immersion in a 200 mV ORP standard solution for 1–2 h. Residual O_3_ concentration was monitored using the indigo colorimetric method (Bader and Hoigné, 1981), which was also used to calibrate the ORP controller. Residual O_3_ concentration, ORP, dissolved oxygen concentration, and water temperature were recorded just after it exited the O_3_ contact chamber. Using previous experience, we adjusted the values to 250, 380, and 600 mV on the ORP unit to achieve an acceptable dose-response performance for residual O_3_ concentration of 0.2, 0.4, or 0.6 mg/L, respectively (Figure 1). A mean O_3_ dose of 0.5–1.5 mg/L was required to overcome the ozone demand of the recirculating water to be transferred into flow to maintain 0.2, 0.4, and 0.6 mg/L through the contact chamber for 5–15 min. Prior to each depuration assay, oysters were subjected to an adaptation period of 4–6 h with untreated ASW inside the depuration tanks to promote oyster activity and increase filtration rates, and shell movements were monitored to confirm that specimens were alive and actively filtering. When residual ozone was reached, 750 oysters (250 in each tank) were ozonated on perforated plastic grills in single overlapping layers suspended 100 mm above the bottom of each tank. Oysters were treated for 6 h in each trial, and experiments were replicated. Oysters were not fed during the experimental period. Depuration was carried out under controlled physicochemical conditions and monitored routinely with a DS5 Hydrolab multiparameter probe for salinity (27.0 ± 2.0‰), dissolved oxygen (90.0 ± 2.0%), pH (8.0 ± 1.0), and temperature (25.0 ± 1.0°C) conditions similar to those found in the MLS (López et al., 2015). ASW was recirculated in a closed pilot-scale seawater system at a flow rate of 10 ± 1 L/min to provide good aeration. Each complete system consisted of a 300 L depuration tank, recirculating submerged water pump, aeration system (Venturi tube), protein skimmer, 1000 mg/h corona discharge ozonator (model 1000BT-12 Enaly, Shanghai, China), closed ASW chamber, and a cooling unit (model C250, Guangdong Boyu Group CO., LTD., Chaozhou, China), mimicking an industrial depuration process.

To study the effect of superchilled storage on *V. cholerae*, fecal coliforms, *E. coli* and *Salmonella* spp. loads in direct ozone depurated shellstock oysters, the most efficient ozone depuration treatments (0.4 and 0.6 mg/L selected according to the results of the tests described above) were carried out. After each depuration trial, oysters were placed in covered plastic bins to prevent drying, pre-chilled at 4°C and then transferred to a chamber superchilled to -1.0 ± 0.2°C for temperature equalization and storage for up to 14 days. Air temperature in the chamber was logged by an automatic logger with an internal sensor and monitored daily. To evaluate the superchilled storage treatment, 200 non-depurated clean fresh oyster samples were placed in covered plastic bins, pre-chilled at 4°C and superchilled stored as the ozonated samples. The day of processing was defined as day 0, and the ozonated oysters were analyzed on days 0, 5, 9, and 14 of storage. Superchilled oyster samples were analyzed on days 0, 1, 5, 9, and 14 days of storage.

### Survival Assessment

Because residual levels of ozone and the superchilled air storage may cause the shellfish to decrease or inhibit normal activity by cessation of mantle and shell movements (Schneider et al., 2009), reducing the effectiveness of the process, survival and pH were monitored as pH is an indicator of death (Aaraas et al., 2004). A 40-oyster sample from the control and from each repetition tank randomly collected was used to assess the survival of oysters after the 6 h depuration and throughout the time of superchilled storage. Another non-depurated fresh 40-oyster sample was used to assess the survival of oysters throughout superchilled storage. Since oysters are sensitive to physical damage, the animals were carefully handled to minimize stress and mortalities. Mortality quantification was registered in each treatment every day during the 14 days of storage and expressed as the percentage of dead oysters relative to the initial number on day zero. The behavioral activity was evaluated by tapping on gaping bivalve shells using the following criteria: shell valve closed (score 0); shell valve merely open but close immediately after tapping (score 1); shell valve open with some flexibility (score 2); shell valve semi-open or remain open with no response to tactile stimulations (score 3), these specimens were considered non-viable or dead and removed, and their number was recorded. On the same days, pH was measured in the oyster meat-intravalvular liquid mixture with a digital pH meter (Orion Research Inc., Cambridge MA, United States) according to Association of Official Analytical Chemists [AOAC] (1990).

### Bacteriological Analyses

During the depuration trials, duplicate samples of 40 oysters were removed for bacteriological analyses from random sites in each tank at 0 (the end of adaptation period and onset of depuration), 2, 4, and 6 h, and throughout superchilled storage at 0, 5, 9, and 14 days. Within 2 h of collection, oysters were shucked, and meats and intravalvular liquids were pooled under aseptic conditions. Oyster samples were analyzed according to the approved method for fecal coliforms, *E. coli*, and *Salmonella* spp. by the Mexican Ministry of Health (NOM-242-SSA1-2009) (Secretaría de Salud [SSA], 2009). To quantify *Salmonella* spp. in raw and ozonated superchilled oysters, 25 g oyster meat and liquor were homogenized with 25 mL tetrathionate broth to produce a 1:10 dilution and pH was adjusted to 7.0 ± 0.2. This dilution was incubated for 6 h at 35°C. 1-mL aliquots were subjected to 10-fold serial dilutions (1:10, 1:100, and 1:1000). 1.0-mL portions of each dilution were spread onto bismuth sulfite (BSA), Hektoen enteric, xylose lysine deoxycholate agar plates for enumeration of colony forming units which were incubated for 24 h at 35°C. Colonies with the characteristic appearance of *Salmonella* on BSA were detected and counted. Suspected colonies were tested on triple sugar iron agar (TSI), citrate, sulfide indole motility medium (SIM) agars, and urea and methyl red Voges Proskauer broths. All agar media were BD Bioxon (Becton Dickinson de México S.A. de C.V., México, México). Afterward, the oxidase test (*p*-aminodimethylaniline) (Becton Dickinson, Franklin Lakes, NJ, United States) was performed on growth from presumptively positive TSI slants from BSA. Presumptively positive *Salmonella* spp. colonies were expressed as CFU/g of oyster meat. The fecal coliforms and *E. coli* data were expressed as Most Probable Number (MPN/100 g).

#### PCR Assays

*Vibrio cholerae* quantification was performed following the MPN-PCR (Most Probable Number-Polymerase Chain Reaction) procedure described by López et al. (2015). PCR assays were performed using specific primers (Sigma-Aldrich QUIMICA S.A. de C.V., Toluca, Mexico) for species and identification of pathogenic genes. DNA of strain CAIM 1406 from the Collection of Aquatic Important Microorganisms (CAIM) ^1^ was used as positive control for the non-pathogenic (*ompW* outer membrane protein) and pathogenic (*chxA* Cholix A toxin) genes (Nandi et al., 2000; Purdy et al., 2010), and strain CAIM 1408 for pathogenic genes (*ctxA*) and the pandemic (O1) (Hoshino et al., 1998). A 100-bp ladder (100–3,000 bp; oxygen) was used as a DNA size marker. Densities of non-pathogenic and pathogenic *Vc* strains was expressed by the Most Probable Number (MPN) method with the 3-tube test series MPN chart corresponding to 95% confidence limits and the results expressed as *V. cholerae* MPN/g of oyster (United States Department of Agriculture [USDA], 2008). The retention rate was determined as follows: retention rate = (bacterial density after time of treatment)/(initial bacterial density) × 100%.

### Statistical Analysis

Most probable number (MPN) chart and formulas were used to identify MPN for each sample (United States Department of Agriculture [USDA], 2008). MPN values for fecal coliforms, *E. coli* and *V. cholerae* counts were log-transformed to normalize the data and homoscedasticity requirements for appropriate analysis of variance. Data were analyzed for significant differences among ozone treatments and days of storage by an analysis of variance (*P* < 0.05). Weighted PCA was carried out in order to gain an overview of the similarities and differences among the single variables. The relationship between microbiological levels of treatments and pH was analyzed by Regression Analysis with Pearson correlation (*P* < 0.05). All evaluated parameters were included in the analysis. All statistical analyses were carried out with *X*LSTAT > 2014.3.02 software (Addinsoft^TM^) with the minimum level of significance set at *P* < 0.05. *V. cholerae* counts of <0.30 MPN/g (non-detectable) were considered 0.15 MPN/g for statistical purposes the effect of superchilled storage on microbiological count.

## Results

### Occurrence of Fecal Coliforms, *Escherichia coli, Salmonella* spp., and *Vibrio cholerae* in Naturally Contaminated Oysters at Harvest

**FIGURE 2:**
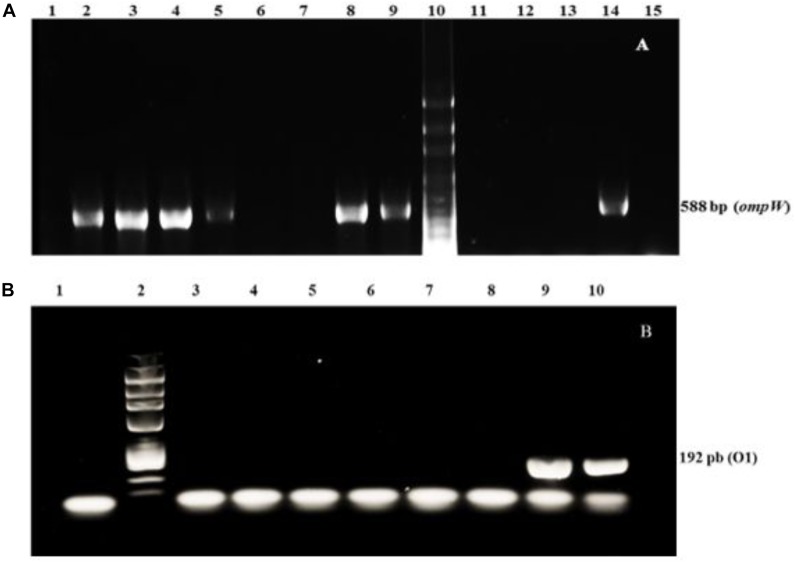
Electrophoretic profiles. **(A)** *ompW* PCR. Lane 1: blank (no DNA); Lanes 2–4, 8, and 9: positive samples; Lanes 5, 6, 7, 11–13: negative samples; Lane 10: 100 bp DNA Ladder; Lane 14: positive control (*V. cholera* non-O1/non-O139, CAIM 1407). Lane 15: negative control (*V. vulnificus*, CAIM 610). **(B)** O1 (*ctxA*) PCR. Lane 1: blank (no DNA); Lane 2: 100 bp DNA Ladder; Lanes 3–7: negative samples; Lane 8: negative control (*V. vulnificus*, CAIM 610); Lanes 9 and 10: positive controls (*V. cholera* O1, CAIM 1408). Numbers on the right indicate the size of the amplification products corresponding to the 588 and 192 bp internal fragments of the *ompW* and O1 genes, respectively.

Tables 1, 3, 5 showed that fecal coliforms and *E. coli* levels in the raw oyster samples used in the experiments were high. Fecal coliforms ranged from 4,100 to 16,000 MPN/100 g and *E. coli* from 1,500 to 3,650 MPN/100 g, placing the sampled oysters in Class B quality, according to European sanitary standards (Council of the European Community [CEC], 2004). Therefore, oysters must be subjected to relay and/or purification. Amplifications of the *ompW* target gene in *V. cholerae* isolates are shown in Figure 2A, indicating the presence of *V. cholerae* non-O1/non-O139, while Figure 2B reveals the absence of *Vibrio cholerae* O1/O139 strains. Tables 2, 4, 5 show the concentration during the dry season of *V. cholerae* non-O1/non-O139 initially present in naturally contaminated oysters in a range of 13.0 to 175.0 MPN/g, while *Salmonella* spp. in the raw oyster samples varied from 2.270 to 3.845 × 10^3^ CFU/g.

**Table 1:** Retention rates and changes in Fecal coliforms and *Escherichia coli* levels in naturally contaminated American oyster (*Crassostrea virginica*) depurated with ozonated seawater.

		Ozone (mg/L)		
Time (hour)	Control^$^	0.2	0.4	0.6
**Fecal coliforms (MPN/100 g)**				
Raw oysters^∗^	16000 ± 2^a,x^	9400 ± 2^a,y^	4450 ± 343^a,y^	4100 ± 838^a,y^
0^†^	8650 ± 1061^b,x^	2950 ± 778^b,x^	300 ± 42^b,y^	1500 ± 283^b,x^
	(54.0)^¥^	(31.4)	(6.7)	(36.6)
2	4450 ± 1344^c,x^	640 ± 14^c,y^	40 ± 28^b,z^	330 ± 7^b,y^
	(51.5)	(21.7)	(13.3)	(22.0)
4	2200 ± 849^b,x^	335 ± 7^c,y^	30 ± 14^b,z^	220 ± 44^b,y^
	(25.4)	(11.4)	(10.0)	(14.7)
6	4450 ± 1344^b,x^	300 ± 42^c,y^	20 ± 2^b,z^	20 ± 2^b,z^
	(51.5)	(10.2)	(6.7)	(1.3)
***E. coli* (MPN/100 g)**				
Raw oysters^∗^	3650 ± 283^a,x^	3500 ± 271^a,x^	3400 ± 165^a,x^	3400 ± 165^a,x^
0^†^	3500 ± 133^b,x^	300 ± 41^b,y^	375 ± 17^b,y^	440 ± 28^b,y^
	(95.9)	(8.6)	(11.0)	(12.9)
2	2500 ± 424^c,x^	280 ± 85^b,y^	30 ± 14^b,z^	30 ± 14^b,z^
	(71.4)	(93.3)	(8.0)	(6.8)
4	1045 ± 360^b,x^	185 ± 21^b,y^	25 ± 7^b,z^	35 ± 14^b,z^
	(29.9)	(61.7)	(6.7)	(8.0)
6	1300 ± 279^b,x^	50 ± 14^c,y^	20 ± 2^b,y^	20 ± 2^b,y^
	(37.1)	(16.7)	(5.3)	(4.5)

*^$^Values are mean densities during depuration with 0.2, 0.4, and 0.6 mg/L. ^∗^Contamination levels in raw oysters before depuration treatment. ^†^Contamination levels after 4–6 h of adaptation period without ozone. ^¥^Retention rates expressed as mean percentage. ^*a,b*^Means with different letters are significantly different (*P <* 0.05) through depuration time within each treatment. ^*x,y*^Means with different letters are significantly different (*P <* 0.05) among treatments within each time*.

### Antibacterial Efficacy of Ozonated ASW Depuration Against Bacteria

The ozone demand of water in the recirculating system before oyster depuration was low, and fecal coliforms and *E. coli* counts of the ozonated ASW after sanitization were low (<0.3 MPN/100 mL). The effect of ozone on fecal coliforms, *E. coli, V. cholerae*, and *Salmonella* spp. loads on American oysters depurated 6 h are shown in Tables 1, 2. The antibacterial efficacies of ozonated ASW against the isolated strains were different. Fecal coliform levels in raw oysters in all ozone depuration treatments decreased significantly (*P* < 0.05) after 2 h depuration. After 6 h depuration, the mean counts decreased significantly (*P* < 0.05) from 9,400, 4,450, and 4,100 MPN/100 g at time zero to 300, 20, and 20 MPN/100 g for 0.2, 0.4 and 0.6 mg/L treatments, representing decreases of 1.0, 1.2, and 1.9 log, respectively. Oysters ozonated with 0.4 mg/L attained a lower fecal coliform count than 0.6 mg/L- ozonated oysters. In contrast, a 0.3 log decrease in control oysters was observed. Initial *E. coli* counts decreased significantly (*P* < 0.05) in oysters after 2 h depuration, and after 6 h the levels decreased significantly (*P* < 0.05) from 3,400 to 3,500 MPN/100 g to 50, 20, and 20 MPN/100 g for 0.2, 0.4, and 0.6 mg/L treatments, representing decreases of 0.8, 1.3, and 1.3 log, respectively. However, no significant (*P* > 0.05) 0.4 log decrease in control oysters was observed relative to oyster samples at time 0. Considering the levels of microorganisms in oysters at time zero, *E. coli* levels decreased 62.9, 83.3, 94.7, and 95.5% after 6 h depuration in control and 0.2, 0.4 and 0.6 mg/L treatments, respectively. According to Table 2, *Vibrio cholerae* non-O1/non-139 loads ranged from 13.0 to 32.1 MPN/g in raw oyster control samples, and no significant (*P* > 0.05) reduction in control sample levels was observed during the depuration process, as retention rate after 6 h was 75%. After 2 h of depuration, *Vibrio cholerae* non-O1/non-139 was not detected (<0.3 MPN/g) with 0.4 mg/L ozone treatment, and a 1.0 log reduction after 0.6 mg/L 4 h- depuration (Table 2). As shown in Table 2, *Salmonella* spp. was not detected in oyster samples after 6 h depuration in 0.4 and 0.6 mg/L ozone treatments. In contrast, initial levels in oyster control samples (2.340 × 10^3^ CFU/g) were slightly reduced during 2 and 4 h depuration by less than 1.0 log_10_ CFU/g and up to 2.110 × 10^3^ CFU/g after 6 h. The decrease in *Salmonella* spp. loads in control oysters after 2 and 4 h depuration was less than 1.0 log_10_ CFU/g compared with 0.4 and 0.6 mg/L treatments in which *Salmonella* was not detected.

**Table 2:** Effect of ozone depuration on *Vibrio cholerae* non-O1/non-O139 and *Salmonella* spp. in naturally contaminated American oyster.

		Ozone (mg/L)		
Time (hour)	Control^$^	0.2	0.4	0.6
***Vibrio cholerae* non-O1/non-O139 (MPN/g)**				
Raw oysters^∗^	13.0 ± 2.8^a^	32.1 ± 0.8^a^	28.5 ± 1.3^a^	19.8 ± 0.9^a^
0^†^	10.0 ± 2.8^a^	26.5 ± 0.7^b^	13.5 ± 0.7^b^	15.5 ± 0.7^b^
	(77.0)^¥^	(82.6)	(47.4)	(78.3)
2	9.5 ± 0.7^a^	14.0 ± 1.4^c^	<0.3^c^	7.6 ± 1.4^c^
	(95.0)	(52.8)		(49.0)
4	7.5 ± 0.7^a^	5.1 ± 0.7^c^	<0.3^c^	<0.3^d^
	(75.0)	(19.2)		
6	7.5 ± 0.7^a^	<0.3^d^	<0.3^c^	<0.3^d^
	(75.0)			
***Salmonella* spp. (10^3^CFU/g)**				
Raw oysters^∗^	2.340 ± 0.800^a^	NA	3.035 ± 0.035	2.270 ± 0.042
2	2.200 ± 0.100^a^	NA	ND	ND
	(94.0)			
4	2.185 ± 0.300^a^	NA	ND	ND
	(99.4)			
6	2.110 ± 0.221^a^	NA	ND	ND
	(90.2)			

*^$^Values are mean levels during depuration with 0.2, 0.4, and 0.6 mg/L. ^∗^Contamination levels in raw oysters before depuration treatment. ^†^Contamination levels after 4–6 h of adaptation period without ozone. ^¥^Retention rates expressed as mean percentage. NA, not analyzed; ND, non-detectable*.

### Effect of Superchilling on Bacterial Load

The most effective ozone depuration treatments (0.4 and 0.6 mg/L) were repeated and ozonated oysters were stored at -1°C, and results are presented in Tables 3, 4. It can be seen in Table 3 that fecal coliforms and *E. coli* counts in raw oysters declined sharply after the 6 h-depuration in both treatments. The combination of ozone and superchilled storage significantly controlled the levels of fecal coliforms and reduced the *E. coli* counts in both treatments throughout the storage time, compared with control oysters. Considering the retention rates during the storage period from day zero, the 0.4 mg/L treatment attained lower fecal coliform levels, although they increased significantly from 50 MPN/100 g on day 0–280 MPN/100 g on day 14. These levels were below the Mexican legal limits (400 MPN/100g). However, clearance of fecal coliforms to acceptable limits was not achieved with the 0.6 mg/L treatment, as the initial fecal coliform load (100 MPN/100 g) on day zero increased significantly (*P* < 0.05) to 975 MPN/100g. Control oyster sample fecal coliform counts increased significantly (*P* < 0.05) at the end of storage, though they were above the Mexican limits throughout the storage period. In contrast, the *E. coli* counts in oysters depurated with both treatments decreased significantly (*P* < 0.05) with time of storage, although in 0.6 mg/L they increased (*P >* 0.05) at the end of storage. Nevertheless, the Mexican legal limits for *E. coli* (230 MPN/100g) were attained in both treatments. Superchilled storage decreased *E. coli* counts significantly (*P* < 0.05) in control oysters as well. As shown in Table 4, *V. cholerae* non-O1/non-O139 counts in superchilled oyster control samples were reduced 1 log on day 9 of storage from 102.0 to 9.4 MPN/g. The maximum reduction of 2 log from 84.0 and 93.5 MPN/g to <0.3 MPN/g was observed on day 5 in oysters treated with 0.4 mg/L and 0.6 mg/L, respectively. However, *V. cholerae* non-O1/non-O139 levels increased to 3.0 MPN/g on day 14 in 0.4 mg/L, and on days 9 and 14 in 0.6 mg/L treatments. Superchilled storage decreased *V. cholerae* non-O1/non-O139 counts by 70% on day 5 in control oysters, while *V. cholerae* non-O1/non-O139 and *E. coli* levels were reduced in 0.4-ozonated oysters by 100.0 and 50%, respectively. The initial levels of *Salmonella* spp. in the control oyster sample (2.11 × 10^3^CFU/g) after 6 h depuration decreased 98% after 14 days of superchilled storage. In contrast, *Salmonella* spp. was not detected in ozonated superchilled oysters.

**Table 3:** Retention rates and changes in Fecal coliforms and *Escherichia coli* levels in naturally contaminated American oyster (*Crassostrea virginica*) depurated with ozonated seawater and superchilled stored at -1°C.

		Ozone (mg/L)	
Time (days)	Control^$^	0.4	0.6
**Fecal coliforms (MPN/100 g)**			
Raw oysters^∗^	8850 ± 1112^a,x^	12600 ± 4808^a,x^	12600 ± 4808^a,x^
0^†^	8500 ± 1061^b,x^	50 ± 13^a,y^	100 ± 38^b,y^
	(96.0)^¥^	(0.4)	(0.8)
5	1500 ± 344^c,x^	50 ± 42^b,y^	330 ± 7^b,z^
	(17.6)	(100.0)	(330.0)
9	2200 ± 849^c,x^	125 ± 2^b,y^	410 ± 44^c,z^
	(25.9)	(250.0)	(410.0)
14	4450 ± 1344^c,x^	280 ± 71^c,y^	975 ± 88^c,z^
	(52.4)	(560.0)	(975.0)
***E. coli* (MPN/100 g)**			
Raw oysters^∗^	2000 ± 356^a,x^	1500 ± 155^a,x^	3400 ± 165^a,x^
0^†^	800 ± 45^b,x^	60 ± 28^b,y^	100 ± 56^b,y^
	(40.0)	(4.0)	(2.9)
5	30 ± 14^c,x^	30 ± 14^b,x^	65 ± 35^b,x^
	(3.8)	(50.0)	(65.0)
9	20 ± 2^c^,^x^	20 ± 2^b,x^	30 ± 14^b,x^
	(2.5)	(33.3)	(30.0)
14	95 ± 106^c,x^	20 ± 2^b,x^	95 ± 21^b,x^
	(11.9)	(33.3)	(95.0)

*^$^Values are mean densities during depuration with 0.2, 0.4, and 0.6 mg/L. ^∗^Contamination levels in raw oysters before depuration treatment. ^†^Contamination levels in oysters after 6 h of ozone depuration. ^¥^Retention rates expressed as mean percentage. ^*a,b*^Means with different letters are significantly different (P *<* 0.05) through storage time within each treatment. ^*x,y*^Means with different letters are significantly different (*P <* 0.05) among treatments within each time*.

**Table 4:** Survival of *Vibrio cholerae* and *Salmonella* spp. in ozonated American oyster (*Crassostrea virginica*) superchilled stored at -1°C.

		Ozone (mg/L)	
Time (days)	Control^$^	0.4	0.6
***Vibrio cholerae* non-O1/non-O139 (MPN/g)**			
Raw oysters^∗^	102.0 ± 11.3^a^	84.0 ± 12.7^a^	93.5 ± 0.7^a^
0^†^	68.0 ± 8.5^b^	63.0 ± 1.4^a^	73.5 ± 2.1^a^
	(66.7) ^¥^	(75.0)	(78.6)
5	20.5 ± 0.7^b^	<0.3	<0.3
	(30.1)	(ND)	(ND)
9	9.4 ± 0.0^c^	<0.3	3.0 ± 0.0^b^
	(14.0)	(ND)	(4.1)
14	3.0 ± 0.0^c^	3.0 ± 0.0^b^	3.0 ± 0.0^b^
	(4.4)	(4.8)	(4.1)
***Salmonella* spp. (10^3^CFU/g)**			
Raw^∗^	2.340 ± 0.800^a^	3.035 ± 0.035	2.270 ± 0.042
0^†^	2.110 ± 0.221^a^	ND	ND
	(90.2)		
5	0.890 ± 0.210^b^	ND	ND
	(42.2)		
9	0.520 ± 0.707^b^	ND	ND
	(24.6)		
14	0.042 ± 0.163^c^	ND	ND
	(2.0)		

*^$^Values are means in control oysters during depuration with 0.4 and 0.6 mg/L. ^∗^Contamination levels in raw oysters before depuration treatment. ^†^Contamination levels in oysters after 6 h of ozone depuration. ^¥^Retention rates expressed as mean percentage. ^*a,b*^Means with different letters are significantly different (*P<* 0.05) through storage time within each treatment. ND, non-detectable*.

Results of ozonated-superchilled oysters were compared with the survival of fecal coliforms, *Escherichia coli, Vibrio cholerae*, and *Salmonella* spp. in naturally contaminated and non-ozonated oysters during superchilled storage at -1°C, shown in Table 5. After 5 days, fecal coliform levels (2,100 MPN/100 g) decreased (*P* < 0.05) but were higher than levels found in control (1,500 MPN/100 g), 0.4 and 0.6 mg/L ozonated oyster samples (50 and 330 MPN/100 g, respectively). The *E. coli* levels in superchilled oyster samples (566 MPN/100 g) decreased (*P* < 0.05) and were higher than those observed in control (30 MPN/100 g), 0.4 and 0.6 mg/L ozonated oyster samples (30 and 65 MPN/100 g, respectively) as well (Table 3). Similar behavior was observed on days 9 and 14. In the case of *V. cholerae* non-O1/non-O139 superchilled storage decreased (*P* < 0.05) by 78% the levels from 175.0 to 38.0 MPN/g, but these counts were higher than those observed in control oyster samples (20.5 MPN/g), and in 0.4 and 0.6 mg/L ozonated oyster (<0.3 MPN/g). Superchilled storage decreased (*P* < 0.05) *Salmonella* spp. levels from 3.845 to 1.370 10^3^CFU/g on day 5 which were higher than those found in control oysters (0.890 10^3^CFU/g). In contrast, *Salmonella* spp. was not detected in 0.4 and 0.6 mg/L ozonated oyster samples (Table 4). The Mexican legal limits for fecal coliforms and *E. coli* (400 and 230 MPN/100 g, respectively) and for *V. cholerae* non-O1/non-O139 and *Salmonella* spp. (absence) in non-ozonated oyster samples were not attained during superchilled storage.

**Table 5:** Survival of Fecal coliforms, *Escherichia coli, Vibrio cholerae*, and *Salmonella* spp. in non-ozonated American oyster (*Crassostrea virginica*) superchilled stored at -1°C.

Time (days)	Fecal coliforms	*E. coli*	*Vibrio cholerae non-O1/*	*Salmonella spp.*
	(MPN/100 g)	(MPN/100 g)	non-O139 (MPN/g)	(10^3^CFU/g)
Raw oysters^∗^	10500 ± 707a	2135 ± 70a	175.0 ± 35.4a	3.845 ± 0.177a
1	10100 ± 1273a	1281 ± 70b	121.0 ± 41.0a	2.715 ± 0.460a,b
5	2100 ± 502b	566 ± 35b	38.0 ± 0.7b	1.370 ± 0.184b,c
9	3600 ± 430b	280 ± 7c	16.0 ± 2.8c	0.795 ± 0.559b,c
14	5300 ± 361c	196 ± 14c	7.2 ± 0.7b	0.072 ± 0.141c

*^∗^Contamination levels in raw oysters before superchilling. ^*a,b*^Means with different letters are significantly different (*P <* 0.05) through storage time within each treatment*.

### Survival

In the present study, most of the ozonated oysters were alive after 14 days of superchilled storage at -1°C. Mortality of superchilled, control oysters and those in both ozonated treatments occurred on day 5; however, survival percentage decreased significantly on days 9 and 14 in control oysters, on day 9 in 0.4 mg/L treatment and superchilled, and on days 5, 9, and 14 in 0.6 mg/L oyster treatment. The cumulative mortality rates after 14 days of storage were statistically different (*P* < 0.05), being mortality of superchilled oysters (22.2%) higher (*P* < 0.05) than 0.6 mg/L O_3_ (7.2%) and 0.4 mg/L O_3_ (5.8%) treatments, and ozonated control oysters (5.6%). These results indicated a greater detrimental effect on superchilled oyster survival. Although loss of mantle fluid was observed, ozonated oysters maintained tight shell lock through treatments with no damage to oyster’ meats. Ozone concentration seems to have a major influence and could explain the lower mortality rate in the 0.4 mg/L ozonated oysters than in those in the 0.6 mg/L treatment. According to Table 6, during superchilled storage pH decreased (*P* < 0.05) in control and ozonated oysters after 6 h depuration. pH values in superchilled oysters decreased (*P* < 0.05) from 6.55 to 6.39 on day 5 of storage, which was lower (*P* < 0.05) than that in control and ozonated oysters. On day 5, first oyster mortality (11.1%) was observed. Moreover, pH values in control oysters decreased significantly (*P* < 0.05) throughout the storage period but not in oysters of the two ozone treatments.

**Table 6:** Changes in pH values in naturally contaminated American oyster (*Crassostrea virginica*) superchilled only and depurated with ozonated seawater and superchilled stored at -1°C.

		0.4 mg/L O_3_		0.6 mg/L O_3_	
Time (days)	Superchilled oysters	Control	Ozonated oysters	Control	Ozonated oysters
Raw oysters^∗^	6.55 ± 0.05a,x	6.55 ± 0.07a,x	6.55 ± 0.07a,x	6.60 ± 0.00a,x	6.60 ± 0.00a,x
0^†^	NA	6.40 ± 0.00b,x	6.40 ± 0.00b,x	6.25 ± 0.07b,x	6.10 ± 0.14b,y
5	6.39 ± 0.01b,y	6.45 ± 0.07a,x	6.50 ± 0.07a,b,x	6.50 ± 0.14c,x	6.55 ± 0.07a,x
9	6.43 ± 0.03a,x	6.30 ± 0.14b,x	6.40 ± 0.00b,x	6.50 ± 0.00a,x	6.50 ± 0.00a,x
14	6.42 ± 0.08a,x	6.40 ± 0.00b,x	6.50 ± 0.00a,b,x	6.40 ± 0.00b,x	6.45 ± 0.07a,x

*^∗^Contamination levels in raw oysters before depuration treatment. ^†^Contamination levels in oysters after 6 h of ozone depuration. ^*a,b*^Means with different letters are significantly different (*P<* 0.05) through storage time within each treatment. ^*x,y*^Means with different letters are significantly different (*P <* 0.05) among treatments within each time. NA, not analyzed (superchilled oysters were not depurated)*.

## Discussion

The presence of fecal coliforms, *E. coli*, and *Salmonella* spp. in oysters is indicative of contamination of fecal origin. They can enter the aquatic environment in oyster-growing areas near the densely populated coast where agricultural and industrial activity is intense. The presence of *Salmonella* bacteria is an important public health issue in Mexico, with high rates of infection reported annually. In 2017, a total of 41,917 cases in Mexico were reported of which 6,116 were in the state of Veracruz (Secretaría de Salud [SSA], 2017). High levels of *Salmonella* (3.56 log CFU/g) like those obtained in our study were reported in naturally polluted oysters (*Saccostrea cucullata*) in India (Jana et al., 2013). India has oceanographic conditions similar to those of Veracruz with tropical and warm seawater areas. *Salmonella* spp. are commonly isolated from seawater in tropical regions and high counts are related to long periods of torrential rainfall, when contamination is transported from source points to the sea via river water (Simental and Martínez-Urtaza, 2008). The Jamapa, Huatusco, Cotaxtla, and Totolapan river plumes that contribute to MLS and water temperature in this tropical area might explain the presence of these pathogens in oysters harvested from this lagoon. Nevertheless, this area is economically important for seafood production and consumption and recreation and the MLS is one of the largest shellfish-producing estuarine lagoons on Mexican Gulf Coast producing and harvesting oysters year-round. Oysters from the MLS supply seafood restaurants and oyster outlets in nearby cities such as Veracruz and Boca del Río. They are also shipped to Cancun, Monterrey, and Mexico City. However, *E. coli* (600 MPN/100 g) and *V. cholerae* non-O1/non-O139 (3.0–28.0 MPN/g) have been isolated in oyster samples from the MLS (Pardío et al., 2010; López et al., 2015). From a public health perspective, consumption of these raw oysters should be considered a potential health hazard.

After 6 h ozone depuration the levels were below the maximum tolerable limits set by Mexican regulations for fecal coliforms (400 MPN/100 g) and *E. coli* (230 MPN/100 g) (NOM-242-SSA1-2009; Secretaría de Salud [SSA], 2009). The Limits for Verification of Depuration Plant Performance from the USFDA National Shellfish Sanitation Program (NSSP) Guide for Fecal Coliform of 20 MPN/100 g (70 MPN/100 g 90th percentiles) (United States Food and Drug Administration [USFDA] and National Shellfish Sanitation Program [NSSP], 2013) were met as well. According to our findings, it is evident that bacteria were not cleared at the same rate. The mechanisms involved in microbial inactivation by ozone are complex and the susceptibility of bacteria to ozone varies among genera, species, and stage of cellular growth. Moreover, microorganisms are inactivated by ozone at different rates, possibly because of differential membrane permeability (O’Donnell et al., 2012). Several studies have reported that fecal coliforms showed considerably less sensitivity to ozone treatments, suggesting that they are more ozone-resistant than the other organisms (Adams et al., 1989; da Silva et al., 1998). Prabakaran et al. (2012) reported that *E. coli* revealed high sensitivity to ozone (0.1 mg/L for 15 min) compared to *Klebsiella pneumoniae*. Thus, the effectiveness of ozone in killing coliforms depends on the specific coliform bacteria. Effectiveness may also depend on other factors, such as locations where bacteria are attached, since ozone mainly reduces surface contamination, and inherent differences in bacterial cell envelope, which is a primary target of ozone activity (O’Donnell et al., 2012).

The depuration results showed that there is a difference between the abilities of *Salmonella* spp. and *E. coli* to survive in oysters. *E. coli* concentration in the control oyster sample was reduced 63% while *Salmonella* spp. decreased 10% after 6 h-depuration. Morrison et al. (2012) found that 15 days after exposure to closed slow flux ASW depuration, an average of 10^3^ CFU/g of *Salmonella* Newport LAJ160311 remained in the oyster meat (*Crassostrea gigas*), while an average of 5.0 CFU/g of *E. coli* ATCC 25922 survived. Relatively few studies focused on quantification of *Salmonella* spp. in ozonated and naturally contaminated oysters or bivalves are available. Çolakolu et al. (2014) reported mean initial *Salmonella* *typhimurium* loads (8.8 ± 0.4 and 6.4 ± 0.6 log_10_ CFU/g) in *Donax trunculus* and *Tapes decussatus*, respectively. *Salmonella typhimurium* was the first strain excreted from both clam species after 66 h of ozone depuration with an open-circuit (6 L/min) at 50 mg/h O_3_. However, little is currently known about how *Salmonella* interacts with marine invertebrates. According to Cox et al. (2016), the *ssrB* regulated effector could contribute to the fitness of *Salmonella* in oysters through regulatory mechanisms which are not currently understood. *ssrB* is required for intracellular survival in vertebrate macrophages. Komanapalli and Lau (1998) studied the effect of short-term ozone exposure at 600 mg/L (1–30 min) on *E. coli* K-12 and observed progressive degradation of intracellular proteins and membrane permeability, cell viability was unaffected, but progressively decreased with longer exposure. In contrast, a *Salmonella* Enteritidis population in distilled water decreased 6 log at 1.5 mg/L of ozone, as ozone treatment disrupted the cell membranes followed by the lysis reaction affecting cell viability (Dave, 1999). In addition to the damage to microbial cell envelopes, ozone may induce mutagenic effects on *Salmonella* *typhimurium*, leading to cell injury or inactivation (Dillon et al., 1992).

The oyster samples reached the legal limit set by Mexican regulations for *V. cholerae* non-O1/non-139 and *Salmonella* spp. of absence per 50 g of oyster flesh after 4 and 6 h-depuration with 0.4 and 0.6 mg/L treatments, respectively. Although the study was conducted during the dry season, when the possibility of high levels of contamination increases, results confirm the effectiveness of the depuration system design. Nevertheless, fecal coliforms and *E. coli* counts did not decrease to zero levels. It has been reported that 0.2% of the originally accumulated bacterial level would likely remain in the shellfish through a 50-h depuration period (Bella et al., 2000). Oysters released *E. coli*, which reached the legal bacteriological limits after 2 h depuration with 0.4 and 0.6 mg/L treatments, while *V. cholerae* non-O1/non-139 limits were reached after 2 h and 4 h depuration with 0.4 and 0.6 mg/L, respectively, indicating that *E. coli* was more sensitive than *V. cholerae* non-O1/non-139. Several ozone depuration studies have reported that naturally occurring bacteria such as *Vibrio* spp. are not likely to be removed effectively due to differential reduction rates of bacteria in depurating shellfish. Croci et al. (2002) reported that experimentally contaminated mussels (*Mytilus galloprovincialis*) showed a reduction of *E. coli* (42%) after 5 h, while *V. cholerae* O1 and *V. parahaemolyticus* declined by 1 log after 24 h depuration with 50 mg/h ozone, remaining almost constant for 44 h. Likewise, Meloni et al. (2008) showed a decrease in *E. coli* counts after 8 h depuration, while naturally occurring *Vibrios* declined at a slower rate (6.7%). Çolakolu et al. (2014) reported that *E. coli* and *Salmonella enterica* subsp. *enterica* serovar *typhimurium* were eliminated in 78 and 66 h, respectively, whereas *V. parahaemolyticus* was present after 72 h in clams (*Donax trunculus* and *Tapes decussatus*), depurated with 50 mg/h-ozone. However, an increase in *Vibrionaceae* species (21%) was observed in Pacific oysters (*Crassostrea gigas*) submerged in ozonated water (0.005 mg/L-2 min) (Rong et al., 2010a). These reports differ from our findings. The results presented here indicate the efficacy of 0.4 mg/L- ozone depuration in reducing 93.3% of fecal coliforms and 94.7% *E. coli* (Table 1), 100% of *V. cholerae* non-O1/non-O139 and *Salmonella* spp. loads (Table 2), in oysters after only 6 h. When depuration periods above 48 h are implemented, bivalve quality may decrease due to the lack of feed during depuration, causing significant economic losses to stakeholders. As depuration may require higher investment and operation costs, oysters should not be killed during processing but kept alive to retain their biochemical and sensorial quality. Thus, it is critical to determine optimal conditions on a species-by-species basis for shellfish microbial depuration to prevent constraints in shell opening. Considering the disinfection efficiency achieved with the depuration system designed for this study, which implied direct application of residual ozone and ozone-produced oxidants to oyster, this approach appears to be a beneficial technology.

The differences observed in the effectiveness of ozone depuration as compared with other reports may also be due to several factors, such as bivalve species, bivalve-species-specific binding potential of microorganisms due to differing mechanisms of persistence, and sensitivity or relative resistance of microorganisms to ozone. Regarding the sensitivity of microorganisms to ozone, free radical activity of molecular ozone can decrease growth (up to inactivation) of specific microbiological species such as *Escherichia coli, Salmonella typhimurium, Staphylococcus aureus, Vibrio parahaemolyticus*, and *Vibrio cholerae* (O’Donnell et al., 2012). Farooq and Akhlaque (1983) observed that lethal concentration of 0.23–0.26 mg/L ozone applied 1.67 min at pH 7.0 and 24°C inactivates *E. coli* and *Salmonella typhimurium* suspended in water, while 0.48–0.84 mg/L of ozone applied 15 min to spring water was needed to reduce *V. cholerae* 95% (Orta et al., 1998). Nevertheless, high growth rates of *V. cholerae* have been observed in seawater treated with high ozone doses (700 mV) (Hess-Erga et al., 2008). Thus, the effectiveness of ozone treatment depends on ozone concentration, length of ozone exposure (contact time), pathogen species and loads, and levels of organic matter (Lee et al., 2008). Several studies have reported that it takes longer to depurate the Eastern oyster (*C. virginica*) of *Vibrio* spp. than of *E. coli* (Murphree and Tamplin, 1991). The lack of depuration efficiency in reducing *Vibrio* levels might be due to the adhesion of *Vibrio* spp. to bivalve’ tissues (Pruzzo et al., 2005). According to these authors, different interactions between soluble hemolymph components and the signaling pathways of the hemocyte bivalve host may be responsible for the persistence of *Vibrio* species within bivalve tissues. This ability of bivalves to exert control over the microbial community in terms of both abundance and biodiversity should be considered, particularly during depuration. The depurating process represents a metabolic effort/fitness that could compromise bivalve’ viability, especially when the suspended food particles are scarce or under stressful conditions (Antunes et al., 2010). Moreover, this ability may explain why artificially contaminated mollusks depurate more rapidly than environmentally contaminated ones (Jones et al., 1991; Schneider et al., 2009). Generally, bacteria are rapidly reduced in shellfish by using seawater only. In this case, the effectiveness of the depuration process depends on the diversity and physiology of the particular mollusk species. Contaminant reduction in the digestive gland is primarily a function of defecation or digestion or both. Several studies (Kueh, 1987; Doré and Lees, 1995) have demonstrated that bacterial reductions may be predominantly influenced by digestive processes, as gut transit times for mollusks are normally rapid. As a prerequisite for decline in coliform number, oysters must pump water through the mantle cavity (Bayne et al., 1976; Gosling, 2003). In this study, contamination levels of raw oysters decreased after 4–6 h of adaptation period due to oyster physiology as microorganisms that were accumulated during previous feeding activities in the alimentary digestive tract of the animal are discharged as part of the fecal material. It is possible that the rates of reduction observed in this study are the actual times required for *Crassostrea virginica* to eliminate fecal coliform and *E. coli* from the digestive tract under depuration conditions and characteristics of the system.

The faster reduction of *E. coli* could induce producers to adopt shorter depuration times, even though high *V. cholerae* and *Salmonella* spp. loads would be present, making the bivalve mollusks a high-risk food and a potential health hazard for consumers. Hence, fecal bacteria and *E. coli* do not adequately predict the comparatively lower rates and levels of *V. cholerae* during oyster depuration or the effectiveness of ozone depuration. This ability of oysters to selectively retain some microbial species has profound public health implications, as depuration practices have been strongly focused on eliminating fecal coliforms. *E. coli* is still used as an indicator of the sanitary quality of bivalve mollusks and their growing areas in México. Therefore, the shellfish industry will be compelled to rely on more efficient post-harvest preservation processes to maintain a commercially viable raw product and protect consumer health, with no significant adverse effects on oyster quality. In recent years, alternative fecal indicators such as fecal anaerobes (genera *Bacteroides* and *Bifidobacterium*, spore-forming *Clostridium perfringens*), viruses [*B. fragilis* phage, coliphages (F-RNA phage)], fecal organic compounds (coprostanol), and nanobiosensors have been increasingly applied (Savichtcheva and Okabe, 2006). It seems that the use of alternative indicators along with conventional fecal markers is promising to identify fecal pollution and associated pathogens.

According to Tables 3, 4, the combination of ozone and superchilled storage significantly controlled the levels of fecal coliforms, reduced the *E. coli* and *V. cholerae* non-O1/non-O139 loads and eliminated *Salmonella* spp. in both treatments throughout the storage time, compared with control oysters. The observed levels were below the Mexican legal limits. The observation that fecal coliforms and *E. coli* population differed during storage could be explained by temperature-induced differences in adaptation and competitiveness within this group of spoilage organisms. The abrupt fecal coliform and *E. coli* mortality on day 5 of storage in control oysters could be linked to the drop-in temperature. These results agree with Hood et al. (1983), who reported that the mean initial fecal coliform count (7.0 × 10^5^ MPN/g) in *Crassostrea virginica* stored at 2°C decreased to 1.7 × 10^5^ on day 7 but increased to 2.5 × 10^6^ MPN/g on day 14, while *E. coli* counts in fresh oysters (2.9 × 10^3^ MPN/g) increased to 2.2 × 10^4^ on day 7 but decreased to 3.0 × 10^3^ MPN/g on day 14. Several fecal coliform strains from environmental sources are psychrotrophic with a minimum growth temperature range of -5 to +5°C (Leclerc et al., 2001). These findings suggest that fecal coliform tolerance and adaptative survival in cold temperatures may be due to the expression of cold-adaptive proteins other than the previously documented major cold shock proteins such as CS7.4 and CsdA reported in *E. coli* (Jones et al., 1992), indicating that non-*E.* *coli* fecal coliforms survive, responding more efficiently to the superchilling storage temperature. These more cold-adapted species, strongly promoted under refrigeration, can reproduce faster in oysters. However, the exact functioning of this cold-adaptive response in several non-*E.* *coli* fecal coliforms remains to be elucidated. In contrast, the analysis of the ribosomal fraction of *E. coli* cells shifted from 37°C to temperatures below 5°C reveals that, during cold-shock, ribosome profiles undergo a severe reduction in polysomes with a concomitant increase in monosomes and cessation of bacterial growth (Hébraud and Potier, 1999). Moussa et al. (2008) reported that the viability of *E. coli* cells decreased from 87% after 10 min to 4% after 71 days at -10°C. The loss of cell viability was attributed to exposure to cold shock which induced membrane damage. Thus, cells could be inactivated by the action of sub-zero temperatures alone.

The combination of ozone depuration and superchilling (-1°C) may have decreased the *V. cholerae* non-O1/non-O139 loads below detection levels. However, the significant increase observed on days 9 and 14 may be related to low-temperature adaptation due to differentially expressed genes involved in the cold shock response of *V. cholerae*. Townsley et al. (2016) reported that the cold shock gene *cspV* of *V. cholerae* is upregulared > 50 upon a low temperature shift from 37 to 15°C. Many of the changes in gene expression are presumably oriented toward overcoming the challenges imposed by cold shock to survive at low temperature. The legal limit of absence in 50 g of oyster meat required by Mexican regulations for *V. cholerae* non-O1/non-O139 was accomplished on day 9 of superchilled storage with 0.4 mg/L treatment, and during 14 days for *Salmonella* spp. in both treatments. Our results differed from those reported by Rong et al. (2010b) who observed that the proportion of *Vibrionaceae* spp. decreased from 20 to 2% on day 5 and was not detected after 60 days in raw non-ozonated Pacific oysters (*C. gigas*) stored at -3°C. *V. cholerae* cells in stationary phase can remain viable for long periods and are even capable of producing disease, as bacteria may retain pathogenicity genes/factors (Pruzzo et al., 2003). Moreover, variability of survivability has been observed among bacterial species. In a study of the effects of freezing on survival of *Salmonella derby, S. typhimurium*, and *E. coli* in Pacific oysters, both species of *Salmonella* were highly sensitive to freezing, and 1% or less survived after 48 h regardless of freezing methods (chest freezer at -23°C, freezer at -34°C, and walk-in freezer at -17.8 °C). *E. coli* was less sensitive to freezing, with a 10–30% survival rate in the oysters after 1 week of storage at -34°C (Digirolamo et al., 1970). In contrast, Gopalakrishna and Shrivastava (1989) reported that *Salmonella paratyphi B*, the most resistant serotype, survived up to 9 months during storage at -20°C. The accumulated evidence suggests that *V. cholerae, E. coli* and *Salmonella* are capable of responding to low temperature stress through production of cold-shock proteins (CSPs) to be cold-adapted to function properly at low temperatures (Jeffreys et al., 1998; Bollman et al., 2001; Phadtare, 2004; Oliver, 2010). Enhanced stress resistance in response to cold shock should draw our attention to the increased risk presented by these pathogens in the seafood industry as low storage temperature is one of the most important processes for controlling the safety of seafood.

A few studies have reported that chilled or superchilled storage combined with ozonated water have been capable of reducing *E. coli, V. cholerae* and *Salmonella* spp. in bivalves over time. Manousaridis et al. (2005) reported a shelf-life of 11–12 days for shucked vacuum-packed and refrigerated mussels (4 ± 0.5°C) ozonated 90 min in an ozone-saturated aqueous solution (1 mg/L) as compared with 8–9 days shelf-life for the non-ozonated sample based on microbiological analyses. Our previous assays carried out during the windy season indicated that ozone depuration (0.2–0.6 mg/L) significantly reduced the low levels of *E. coli* and the isolation of *V. cholerae* in oysters during the superchilled storage period (Pardío et al., 2010). Rey et al. (2012) reported that storage of oyster (*Ostrea edulis*) in ozonated slurry ice (0.2 mg/L ozone) at 0°C ± 2°C provided better control of *Enterobacteriaceae*, with significant (*P* < 0.05) differences being observed between ozonated slurry ice oysters which levels decreased from 1.63 CFU/g on day 1 to 0.90 CFU/g on day 6 of storage and non-ozonated flake ice which levels increased from 1.24 CFU/g to 2.39 CFU/g on day 6 of storage. Ozone- superchilled storage applied to fishery products has been studied as well. Jianbing et al. (2013) reported that superchilling (-1.2°C) combined with ozonated water (1.8 mg/L) had a remarkable effect reducing the aerobic plate count of pomfret fillets. Recently, Bono et al. (2017) found that superchilled storage at -1°C improved the antimicrobial activity of 0.3 mg/L-ozonized slurry-ice during European anchovy (*Engraulis encrasicolus*) and sardine (*Sardina pilchardus*) postharvest preservation, reducing *Enterobacteriaceae* at below 1 log CFU/g throughout superchilled storage.

According to Table 5, superchilled storage at -1°C significantly decreased the levels of fecal coliforms on day 5 and those of *E. coli* on day 1. As previously mentioned, fecal coliform tolerance and adaptative survival in cold temperatures have been observed. The initial level of *Vibrio cholerae* non-O1/non-O139 in non-ozonated oysters on day 5 (175.0 MPN/g) of superchilled storage decreased 78.3% (38.0 MPN/g). These results are similar from those reported by Rong et al. (2010b) who observed that the proportion of *Vibrionaceae* spp. decreased from 20 to 2% (90%) on day 5 in raw Pacific oysters (*Crassostrea gigas*) stored at -3°C. Meanwhile, *Salmonella* spp. counts decreased 35.6% from 3.845 to 1.37 10^3^CFU/g. Gopalakrishnan et al. (2016) isolated *Salmonella* spp. and *Vibrio cholerae* from the various parts of *Rastrelliger kanagurta* (Indian mackerel) stored at -5°C for 5 days, with a microbial load of 6.1 × 10^4^ CFU/g. Thus, given the antimicrobial effect observed in ozonated oysters, there is a beneficial effect of combining ozone depuration and superchilled storage at -1°C on the microbiological safety of oysters. Our results suggest an additive effect of superchilling and ozone pre-treatment in altering microbial integrity and survival on oysters, although some strains may be intrinsically more resistant to cold shock injury than other strains. Several studies have indicated that the residual ozone concentration is greatest at low temperature (O’Donnell et al., 2012). In this context, superchilling storage may facilitate the ozone decomposition mechanism, improving oxidation of membrane glycoproteins and/or glycolipids and the inactivation of microbial cells. From this perspective, controlling the growth of these bacteria through ozonation may be important to improve preservation of oysters during superchilled storage. Improvements in the microbial safety of oysters can have an important economic impact by reducing losses, improving marketability and ensuring public health. In this regard, ozone depuration combined with superchilled storage improved the microbial safety of oysters by a synergic effect.

The mortality rate observed in this study was lower than the 52.5% reported by Maziero and Montanhini (2015) for mangrove oysters (*Crassostrea brasiliana*) after 15 days of storage at 5°C. Although no reports of mortality percentage for ozonated oysters stored at superchilled temperatures were found, it has been reported that eastern oyster *Crassostrea* *virginica*, an eurythermal suspension-feeding bivalve, is able to tolerate a minimum temperature of -2°C (Pernet et al., 2008). During long-term air exposure at cold temperatures oysters can depress metabolic rate and respire anaerobically and are able to survive without available oxygen and seawater. A survival rate of 35.7% after 50 days of air exposure at 4°C has been reported, and the accumulation of acidic end-products and a decline in pH (6.92 to 6.65) were observed within 2–3 days (Kawabe et al., 2010). The decrease in pH of the live bivalves represents the beginning of deteriorative processes that accelerate postmortem. It has been reported that oysters (with liquor) were classified as being of good quality if their pH ≥ 6.0 (Schneider et al., 2009). Intracellular pH is one of the important factors controlling metabolic rate. Moreover, changes in pH are related to shell movement. During shell closure, pH decreases gradually, suggesting an initial reliance on anaerobic metabolic pathways to sustain life (Kawabe et al., 2010). Demonstration of stable pH during storage indicates maintenance of extracellular pH to maintain respiratory gas exchange, but when death occurs the pH rapidly falls. pH is therefore a clear indicator of death (Aaraas et al., 2004). According to our findings, superchilled storage of oysters did not cause a decline in survival ability.

**FIGURE 3:**
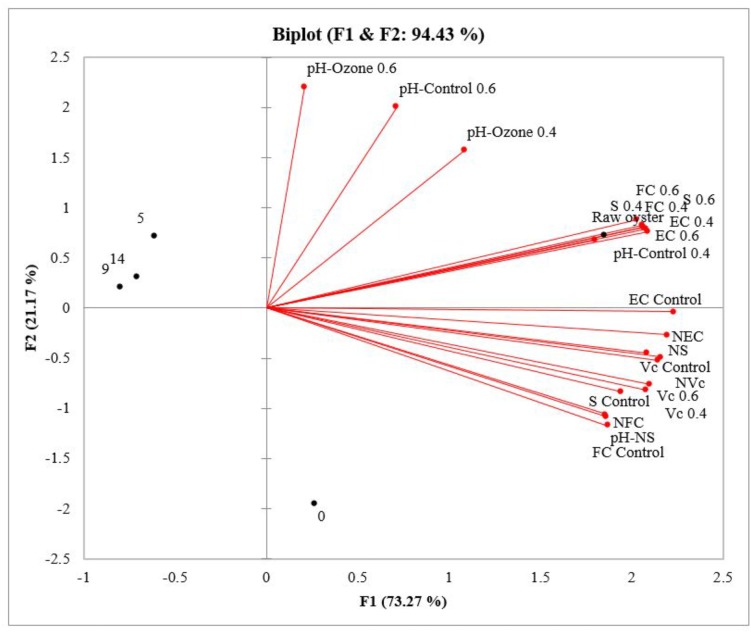
Principal Component Analysis (PCA) projections of scores and loadings for the first two principal components for the analysis of densities of fecal coliforms (FC), *E. coli* (EC), *Salmonella* spp. (S), and *V. cholerae* non-O1/non-139 (Vc) and pH in the raw oyster and control samples, nonozonated- superchilled (N), ozonated- superchilled (0.4 and 0.6 mg/L), and the time of superchilled storage (0, 5, 9, and 14 days). Variables with vectors projected in the same plane may be considered as positively correlated.

According to PCA analysis (Figure 3), the two principal factors described 94.43% of the variation between variables. The first factor, representing 73.27% of the total variation, described primarily the effect of superchilled storage on microbiological count. Accordingly, 68.4% of the variation was observed on raw oysters, and during the superchilled storage 76.5% on day 0, 10.2% on day 5, and 12.8% on day 9. In raw samples, high fecal coliforms, *E. coli*, and *Salmonella* spp. levels were related to pH. On day zero the microbial loads of fecal coliforms, *E. coli, Salmonella* spp. and *V. cholerae* non-O1/non-139 decreased significantly in non-ozonated- superchilled samples, indicating the effect of storage. However, *V. cholerae* levels decreased in ozonated- superchilled samples, indicating the effect of both treatments. Pearson correlation suggested a strong positive relationship between the microbiological levels of fecal coliforms and *V. cholerae* non-O1/non-O139 in non-ozonated- superchilled samples and pH (*r* = 0.976, *p* = 0.05; *r* = 0.910, *p* = 0.05, respectively). Thus, to improve the microbial safety of oysters it is essential to understand the physiology of bivalves during post-harvest storage, as oysters are traded as live animals.

The latest market reports in 2016 reflect an increased trade in oysters, totaling about 70,000 tons, with an estimated price of US$ 20.6 by dozen, and $US 3.3 each (Food and Agriculture Organization [FAO], 2017). According to Chen et al. (2017) an oyster industry may be an economically viable pursuit if a minimum selling price of US$1.35 per oyster can be achieved. In México, the current market price for locally grown non-depurated oysters is US$0.10 per oyster (Comisión Nacional de Acuacultura y Pesca [CONAPESCA], 2017). Considering the depuration process costs plus the cost of oysters (without the superchilling cost), we estimated that the selling price per shellstock oyster would be approximately US$0.20 at the depuration facility, US$0.44 in the central seafood wholesale market, US$0.73 in local restaurants, and up to US$1.60 in restaurants in México City, Monterrey, and Cancún. Although these prices will increase when superchilling process costs are considered, superchilled depurated oyster production would still be profitable as in recent years exports of chilled and frozen bivalves have grown worldwide (Food and Agriculture Organization [FAO], 2016).

## Conclusion

The results of this study demonstrate that *E. coli* is eliminated more rapidly than *V. cholerae* and *Salmonella* spp. from the gut tissue of the tropical oyster *Crassostrea virginica* and thus, is an inadequate microbiological quality index as it is unable to demonstrate general depuration capacity. Ozone depuration for 6 h at 0.4 mg/L enables the efficient reduction of fecal coliforms, *E*. *coli, Salmonella* spp. and *V. cholerae* levels in oysters, below the maximum tolerable limits set by Mexican regulations. Superchilled storage at -1°C improved disinfection efficiency and microbiological quality of ozonated oysters at 0.4 mg/L for up to 9 days. During the storage period the 0.4 mg/L treatment attained lower fecal coliform and *E. coli* levels, reduced 99.5% of *V. cholerae* non-O1/non-O139 counts, and *Salmonella* spp. was not detected. Future studies are still required to assess the efficacy of this process in reducing pathogenic *V. parahaemolyticus* and *V. vulnificus* naturally accumulated in oysters. The absence of ozone depuration resulted in higher levels of fecal coliforms, *E. coli*, and *V. cholerae* non-O1/non-O139 in oyster samples during superchilled storage than those observed in control, 0.4 and 0.6 mg/L ozonated oyster samples. Superchilled storage only decreased *Salmonella* spp. levels in non-ozonated oysters while this bacterium was not detected in ozonated oyster samples during superchilled storage.

Combining the direct-ozone depuration pretreatment and superchilled storage has a synergistic effect and several processing advantages. The depuration time is shorter, the oyster survival is not adversely affected, and the microbiological safety of oysters improves with an acceptable safe level for human consumption up to 9 days. The use of superchilled storage at -1°C of fresh ozonated shellstock oysters is a promising technology but needs further optimization since *V. cholerae, E. coli* and *Salmonella* spp. in cold-stored oysters could potentially survive and remain as a significant health risk for raw oyster consumers. This process combination appears to be a potential technology, holding both practical and economic interest for marketing strategies, and improving the profits of local producers. It may thus represent a viable option for the oyster industry to preserve raw oysters, ensuring microbial safety throughout the supply chain to protect public health.

## Author Contributions

KL and VP contributed conception and design of the study and wrote the manuscript. SR contributed conception and design of the study. VS, IR, and RU performed the analytical techniques. VP, AF, and DM performed the statistical analysis. All authors contributed to manuscript revision, read and approved the submitted version.

## Conflict of Interest Statement

The authors declare that the research was conducted in the absence of any commercial or financial relationships that could be construed as a potential conflict of interest. The reviewer AA and handling Editor declared their shared affiliation.

## Footnotes

1 www.ciad.mx/caim
